# Functionally-explicit sampling can answer key questions about the specificity of plant–microbe interactions

**DOI:** 10.1186/s40793-022-00445-x

**Published:** 2022-10-11

**Authors:** Suzanne M. Fleishman, David M. Eissenstat, Terrence H. Bell, Michela Centinari

**Affiliations:** 1 Department of Plant Science, The Pennsylvania State University, University Park, PA 16802 USA; 2 Department of Ecosystem Science and Management, The Pennsylvania State University, University Park, PA 16802 USA; 3 Department of Plant Pathology and Environmental Microbiology, The Pennsylvania State University, University Park, PA 16802 USA; 4 Graduate Program in Ecology, The Pennsylvania State University, University Park, PA 16802 USA

**Keywords:** Rhizosphere, Microbiome, Root function, Heritability, Root traits, Specificity, Absorptive roots, Review

## Abstract

The rhizosphere is a nexus for plant–microbe interactions and, as a host-structured environment, a location of high activity for distinct microbes and plant species. Although our insights into this habitat have exploded in recent years, we are still limited in our ability to answer key questions about the specificity of these root-microbial relationships. In particular, it can be difficult to confirm or reject microbiome heritability in many plant systems and to pinpoint which microbial taxa are key to plant functioning. Like other host-structured environments, the rhizosphere is structurally, chemically, and biologically complex, driven largely by differences in root anatomy, location, and function. In this Correspondence, we describe a review of 377 “rhizosphere microbiome” research papers and demonstrate how matching a sampling method to the biological question can advance our understanding of host-microbe interactions in a functionally heterogeneous environment. We found that the vast majority of studies (92%) pool all roots from a root system during sampling, ignoring variation in microbial composition between roots of different function and limiting insight into key root-microbial relationships. Furthermore, approaches for removing root-associated microbes are highly variable and non-standard, complicating multi-study analyses. Our understanding of the strength and nature of host-microbe relationships in heterogenous host-microbiome environments can be clarified by targeting sampling to locations of high interaction. While the high complexity of the rhizosphere creates logistical challenges, we suggest that unambiguous language and refined approaches will improve our ability to match methods to research questions and advance our understanding of the specificity of plant-microbial interactions.

**Supplementary Information:**

The online version contains supplementary material available at 10.1186/s40793-022-00445-x.

## Introduction

The rhizosphere is considered a global hotspot for microbial activity and a nexus for the plant–microbe interactions that shape plant health and productivity [1]. The rhizosphere was originally defined by Hiltner in 1904 [2, as cited in 3], and research on the structure and composition of the rhizosphere has led to various definitions, but it is generally considered to be the root-adjacent region in which microbes and roots actively engage with one another [1–11]. Microbial growth and activity are stimulated in the rhizosphere by exudates and other rhizodeposits produced from individual roots, which can either attract or repel particular microbial taxa, each with neutral, positive, or negative implications for plant health and productivity [1, 4, 6, 12]. The microbial occupants of the rhizosphere are highly influenced by environmental conditions and microbe-microbe interactions [12]. Yet, as a host-structured habitat, microbial composition is also structured by plant species and these plant-microbial relationships are the focus of this Correspondence [12]. If these relationships are identified and described, it has the potential to inform our understanding of plant-microbial relationships that promote plant functions, with positive applications in agriculture and other managed plant ecosystems [7–9].

The importance of plant-microbial relationships in the rhizosphere is widely recognized, but until approximately a decade ago research techniques limited efforts to comprehensively characterize microbes in the rhizosphere [5, 8, 13]. Technical advances have led to a recent flush of studies that aim to define key microbes for plant health and ecosystem function. Consequently, we have substantially improved our understanding of certain genotype-level plant-microbial pairings (e.g., legume-rhizobia systems). Controlled studies have identified plant signals involved in recruiting or supporting microbes that promote plant function [14], while large, multi-faceted field studies have allowed detection of microbes that may be important to particular plants under real-world conditions [15]. Despite this progress, we are far from optimizing our ability to assess the specificity of plant-microbial relationships across systems and environmental conditions [12, 16].

With the rapid growth in research on the rhizosphere environment, it is not surprising that methods have been adapted ad hoc without consideration if sampling methodologies are sufficient for this new research frontier. Current approaches are coarse and have been able to detect strong signals from the soil and plant environment, but accounting for the underlying spatial and developmental heterogeneity of the rhizosphere habitat should bring us closer to an understanding of reality [12, 17]. Like other host-microbe systems, the “playing field” in which interactions occur in the rhizosphere is not homogeneous and individual roots within a root system can vary drastically in important functional traits, including exudation rates, nutrient uptake rates, and respiration [18]. Rhizosphere studies typically capture net estimates of microbial populations by pooling roots within and across root systems, which is effective for research questions regarding impacts on the rhizosphere for a root system *on average*; however, a significant tradeoff of this approach is masking heterogeneity within root systems that is important for plant function.

In research on the aboveground portions of a plant, tissues are regularly separated based on morphology and function, likely due to the obvious visual differences, such as those between stems and leaves. In contrast, individual root segments within a root system may visually appear similar despite drastically varied functions. Consequentially these roots are typically pooled together, dulling interactions that are likely to be specific to roots with particular roles. For instance, absorptive capacities of roots are typically strongest in root tips and terminal roots, with increasing distance from the terminal roots, individual roots become thicker and increase in traits related to resource transport or storage [18, 19]. Given that nutrient uptake is one of the main microbially-mediated processes that can promote plant health, we may expect substantial host-microbe interaction along roots dedicated to soil-resource uptake, such as root tips or roots of lower branching order [20]. In contrast, we would not expect the same degree of interaction along roots that are often primarily responsible for resource transport within the plant and can represent up to 25% of lateral roots in a woody root system [21]. Yet absorptive and transportive fine roots (i.e., less than 2 mm in diameter) are nearly always sampled together.

In this Correspondence, we propose that explicitly considering root system heterogeneity will improve our ability to characterize the specificity of plant–microbe interactions across widely divergent plant types and environmental conditions. Importantly, refined methods that are better matched to research questions will allow more sensitive detection of:

- Heritability in microbial recruitment.
- Identification of the key rhizosphere microbes that shape plant health.

We first discuss how root functional heterogeneity can impact microbial recruitment and then describe our systematic review of current sampling approaches, while highlighting their limitations. Finally, we suggest future directions to better match methods to research questions in rhizosphere microbiome research.

## Root-associated microbes exist in a heterogeneous environment

Root systems are complex, including thousands of roots of varied function, each interacting uniquely with the surrounding soil [18, 19]. Estimating the functions of individual roots can be done by assessing various root traits, which can be visually estimated based on anatomy or morphology (e.g., root color), or pinpointed through detailed physiological measurement (e.g., N-uptake rate) [18]. Traits that relate directly to the profile and concentration of root exudates have some of the strongest implications for microbial concentration and activity in the rhizosphere, but these traits are unfortunately difficult to measure accurately, especially with the high replication that is often required for field experiments [22]. As a result, the degree of root-to-root variation in exudation remains uncertain, but evidence suggests that we should expect massive differences, particularly since some roots are primarily involved in substance transport to other plant organs, while others are targeted to acquired water and nutrients, and exchange organic compounds with the surrounding soil environment, including with microbes.

**Fig. 1:**
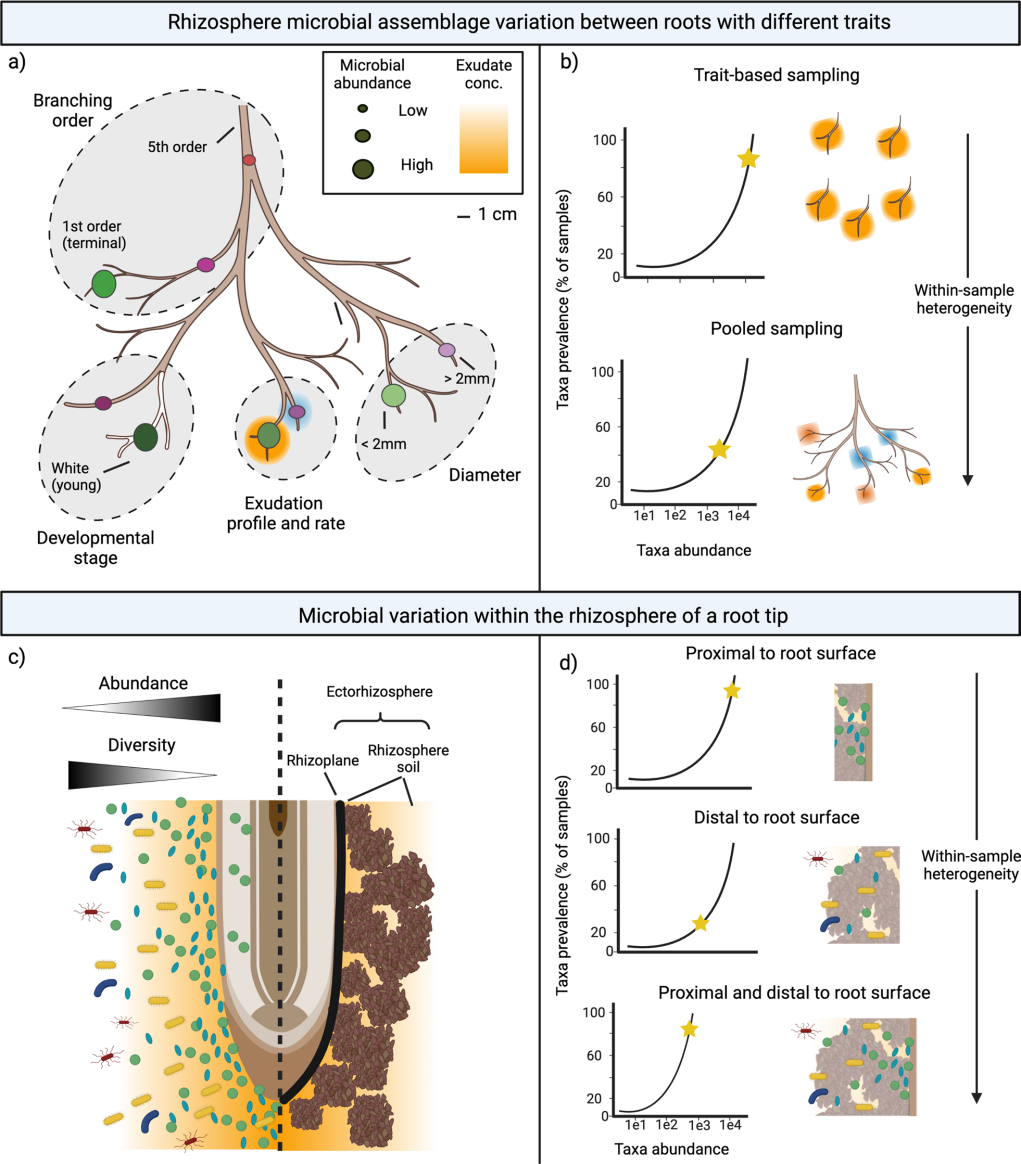
**a** Hypothesized differences in microbial abundance and composition for select root traits that vary in a root system. Within each grey ellipse, circle sizes represent differences in microbial abundance and colors represent differences in composition. **b** Prevalence plots denoting functionally explicit sampling (top) or pooled sampling of a root system (bottom). Black lines denote a taxon’s relative abundance averaged across all samples (x axis) versus percentage of samples where the taxon is present (y-axis). The yellow star within the plots represents changes in locations on prevalence plots of a putative microbe of interest depending on the sampling approach. **c** Conceptual model of the rhizosphere region of a root tip. The left portion of the figure shows changes in microbial abundance and diversity based on distance from the root surface; the right portion of the figure displays the locations of rhizosphere compartments, including the rhizoplane (root surface) and rhizosphere soil. **d** Prevalence plots denoting rhizosphere removal methods that target a region close-to (top), a region distant-from (middle), or a broad region from (bottom) the root surface. Black lines denote a taxon’s relative abundance averaged across all samples (x axis) versus percentage of samples where the taxon is present (y-axis). The yellow star within the plots represents changes in locations on prevalence plots of a putative microbe of interest depending on the sampling approach. Throughout all figures, colored shading around roots represents exudate concentration (conc.) with different colors denoting different exudate compositions and darker shading referring to higher concentrations of exudates in comparison to lighter shading. Figure created with BioRender.com

There is growing evidence that differences in root function, as estimated by morphology, do in fact lead to heterogeneous structuring of rhizosphere microbial composition. In woody perennial root systems, young, white, low branching order (i.e., terminal roots are first order), and smaller diameter roots may have higher metabolic rates and acquire more resources (Fig. 1a) [18, 19]. These active roots may be more attractive to particular microbes and there is evidence of higher microbial abundance and/or distinct microbial composition on roots that are low order [20, 23, 24] and smaller diameter [25], in comparison to other roots in the root system. We also see microbial composition impacted by root type and/or portion of the root system in herbaceous plants, including maize [26, 27] and flowering tobacco [28]. Beyond these aforementioned studies, our review did not present others that have directly compared the microbiomes of root types within a root system, yet this early evidence suggests that roots with distinct functions create unique rhizosphere environments, leading to differentiated microbial assemblages for each root type within a root system (Fig. 1a).

Even across the rhizosphere of a single root, there is evidence of high spatial heterogeneity in microbial composition. With increasing radial distance from the surface of a root tip, microbial abundance decreases, but microbial diversity increases (Fig. 1c) [9]. This means that the locations proximal to or on a root surface (i.e., the rhizoplane) are a distinct and high-abundance microbial habitat. This is suggested to be the result of a higher host-imposed selection close to the root than locations in the soil further away. The plant exudates responsible for recruiting and supporting microbes that are beneficial for plant function are the most concentrated close to the root surface [1, 29]. Thus, we would expect that for a given plant genotype, the specificity of plant–microbe interactions are most detectable in the region closest to the root, although we acknowledge microbe-microbe interactions can also shape the heritability of plant–microbe relationships.

## The consequences of coarse sampling approaches

Clearly, this underlying heterogeneity within root systems, like other host-structured environments, has consequences for microbial distribution and host-microbe interactions [17]. So, to what extent have coarse sampling methods limited our current understanding of rhizosphere microbiomes? Here, by summarizing current research methods and discussing their implications for studies of microbiome heritability and identifying key taxa, we show the limitations of current research approaches and ways to move forward to better address research questions.

**Fig. 2:**
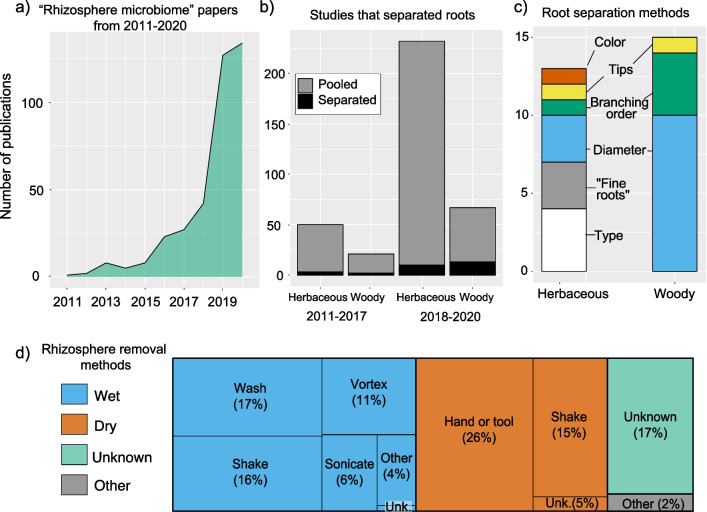
**a** Number of publications per year between 2011 and 2020 for 377 publications found with “rhizosphere microbiome” search terms. **b** Number of publications investigating woody and non-woody roots that separated the root systems based on a root trait at the time of sampling for two time periods. Total bar height represents the total number of studies; black bars represent the number of studies that separated roots (8% of the total) for herbaceous and woody root systems within two time periods. Studies that examined both plant types (n = 7) were excluded from the figure. **c** Stacked bar chart displays the number of papers that separated roots before removing the rhizosphere for both woody and non-woody plants. Colors in each bar represent the root trait used to separate roots. **d** Tree plot of rhizosphere removal methods with box area proportional to the percentage of papers using each removal method. Colors depict overarching four categories of methods: wet (blue), dry (orange), unknown (no information provided on the method; green), and other (gray). Subcategories are indicated by text within boxes for wet and dry methods. The unknown (unk.) subcategories refer to studies which provided sufficient information to establish whether a method was wet or dry, but insufficient information to determine the specific approach

Through a systematic review we aimed to address two important questions: (1) are rhizosphere microbiome sampling methods standard across studies and, if not, which methods are most popular? (2) Do rhizosphere microbiome studies consider heterogeneity across root systems and within the rhizosphere environment? We searched Web of Science for “rhizosphere microbiome” and excluded papers that were conference proceedings, reviews, or did not include examination of rhizosphere microbiota. Papers were evaluated and categorized in 11 areas based on experimental design and methodology (Additional file 1). Our list of papers was periodically updated from July 2019 through June 2021 with a chosen end publication date of December 2020 to give sufficient publications and capture yearly average publication rates; this resulted in 377 papers published from 2011 – 2020 (Fig. 2a).

### Coarse sampling approaches dominate in rhizosphere microbiome research

Since 2011, there has been a drastic increase in “rhizosphere microbiome” publications (Fig. 2a). Yet, 92% of studies did not use functionally-informed sampling of root systems when examining rhizosphere microbiomes, with studies more common on herbaceous than woody plant species (76% vs. 24%; Fig. 2b). Over time, there have been contrasting shifts in the proportion of papers using functionally-informed sampling for herbaceous and woody species (Fig. 2b). When comparing an early, low-publication period of time (2011–2017) to a more recent period with relatively high publication rates (2018–2020), the percentage of studies with functionally-informed sampling nearly doubled (10% to 19%) for publications focused on woody plants, but slightly decreased (6% to 4%) for publications focused on herbaceous plants (Fig. 2b). In addition to being less likely to separate roots based on function, studies on herbaceous plants were also less likely to sample roots to a standard soil depth, in comparison to woody plants (18% and 56%, respectively; Additional file 1). The lower research rate but higher attention to heterogeneity for woody plants may be reflective of both greater difficulty of collecting representative root samples and a greater need for root separation methods for woody plants [12, 19]. Due to their architecture and perennial nature, woody root systems have greater complexity of root morphology, root developmental stage, and rooting depth than what is found in herbaceous plants [12]. For these reasons the increased attention to both root functional heterogeneity and rooting depth in woody plants is promising, yet the predominance of pooled sampling approaches overall has likely had consequences for research conclusions thus far.

Important information is lost with the common method of pooling all roots within a sample. Consider a highly heritable and functionally important microbial type that is only of high relative abundance near young and highly active roots (Fig. 1b). Studies use different approaches to identify key or “core” taxa for a particular plant species or genotype [30, 31] but a common and highly-simplistic approach is to screen a large number of genotypes and environments and determining microbial taxa that are the most prevalent (i.e., occur in a high number of samples) and of high relative abundance (e.g., [29, 32]). When all roots are pooled regardless of root function, this taxon’s importance may be obscured by the high representation of taxa associated with roots that have a greater surface area and different functions within the root system (e.g., structural roots), resulting in false negatives. However, if the roots are sampled and pooled with a functionally-informed approach (e.g., absorptive roots), this noise can be dramatically reduced, allowing the key taxon to be identified more frequently. This would allow its relative abundance to better reflect its relative abundance at key sites of host-microbe interaction rather than within the root system as a whole. The consequences of altering a taxon’s relative abundance in the initial sample will then extend to and impact many common microbiome analytical approaches, including univariate alpha diversity approaches, multivariate beta-diversity approaches, and pairwise testing of the differential abundance of individual taxa.

This issue can be largely resolved by matching sampling schemes to research questions, but there are tradeoffs between functionally-informed sampling and measurement feasibility. In studies that did separate roots by type, diameter was the most commonly used approach for separation and all methods used were based on morphological traits (Fig. 2c). These so-called “soft traits”, including root order, diameter, color, or type, are simple to identify visually, but do not directly relate to root function in the same way as “hard” traits, which are more challenging to measure (e.g., exudation, N-uptake rate, respiration) [18]. Analyzing many “hard” traits comes with an extensive time lag, or requires destructive sampling, which damages roots and/or compromises the rhizosphere in the measurement process. This means it may not be feasible to classify and separate roots ahead of sampling. Despite these tradeoffs, any efforts to reduce variability or noise in root sampling can help to add nuance to our currently coarse understanding of rhizosphere microbiomes. These efforts will be constrained by the specific plant under study and limitations of a research site (e.g., sampling time or sample size). Extensive information on root functional traits and the tradeoffs associated with methods of root sampling are outlined in Freschet et al., (2021a) and (2021b) and can assist researchers in identifying the best sampling approaches [18, 33].

### Rhizosphere removal methods lead to capture of different microbial populations

While we found clear evidence that pooling all roots in a sample is the most popular root sampling approach, we did not find a single popular method for removing rhizosphere soil. A surprisingly high proportion of studies (17%) do not report methods in a reproducible manner and make the statement “rhizosphere soil was removed” with no reference to another study’s methods (i.e., the unknown category; Fig. 2d). Of the studies that provided information on their approach, we divided rhizosphere removal approaches into two larger categories with somewhat similar proportions of studies in each: wet (55%) or dry (43%) (Fig. 2d). Following root sampling, wet methods agitate roots with a sterile liquid to remove adhering soil and microbes, whereas dry methods may remove soil by agitation without a liquid or fall into the broad category of “hand or tool” which includes a multitude of various methods (e.g., “scraped off with disposable spatulas”, “removed with gloved hand”).

Overall, there is drastic variation in the exact removal approach (from 6 to 26%) and this variation has consequences for the microbes that are captured and deemed important. For example, if one study samples the rhizosphere by root sonication in buffer while another shakes roots without liquid, these two studies strongly differ in the region of the rhizosphere sampled. Investigations consistently report differences in microbiomes between bulk soil, root associated soil removed by vortex (i.e., rhizosphere), and a subsequent root-associated fraction removed by sonication (i.e., rhizoplane) [29, 34, 35]. By these definitions, the study using sonication is presumably capturing the entire ectorhizosphere while the study using a vortex is only capturing the microbes in outermost portions of the rhizosphere soil [1, 29]. While in some cases these subtle differences in sampling may not lead to consequences for answering research questions, in others it hinders cross-study comparisons and analyses. If a particular taxon is highly recruited and stimulated by plant exudates, it is presumably most abundant close to the root surface and may be best captured in high proportions by sonication (Fig. 1d). Other methods of removing the rhizosphere could obscure this particular taxon, producing false negatives when attempting to identify key microbes as prevalent or abundant, preventing a cohesion in findings between two studies simply due to rhizosphere removal methodologies. Online sequence databases and meta-analyses are populated enough to facilitate future broad-scale research on rhizosphere microbiomes [36]; however, these potential false negatives in current and nonstandard sampling methods likely hinder multi-study compilations to isolate key or heritable taxa.

Establishing a standard methodology for sampling the rhizosphere in different experimental contexts could address these inconsistencies. During our review process, we noted that since 2018, a few papers tended to be cited more than others for their rhizosphere removal methodologies, including Edwards et al. (2015) [29] and Lundberg et al. (2012) [34], which both vortex roots to remove rhizosphere, then sonicate roots to remove any remaining rhizosphere or surface-adhering microbes (i.e., rhizoplane). These research papers were published in notable journals (*Proceedings of the National Academy of the Sciences* and *Nature*, respectively) with detailed protocols for sampling the rhizosphere published separately, facilitating wide readership and adoption of their practices. While these two papers were noted to be explicitly cited in several studies, we did not notice a concerted trend towards a most popular methodology over time and “hand or tool” was the most popular method overall. At this point in time, methodological research is not available to evaluate the differences in results between these well-cited protocols in comparison to some of the “dry” removal methods that are most popular. Yet, current evidence would suggest that vigorous agitation methods that remove portions of the (ecto)rhizosphere closest to the roots (i.e., sonication; [7, 34]) may be most appropriate for research questions focused on plant-microbial interactions and that less vigorous methods capturing distant portions of the rhizosphere (e.g., dry shake) may be most appropriate for questions related to plant-soil feedbacks. Here we are suggesting that a concerted effort to thoroughly consider the consequences of these removal methods is critical for standardizing operational definitions of the rhizosphere region and reducing the application of multiple, potentially incomparable, methods.

## Conclusion and future directions

Accurately estimating plant–microbe specificity is key to understanding degree of microbiome heritability across plant types and environmental conditions, as well as to identifying taxa that are essential to plant function. The challenges to accurate and standard measurement of the rhizosphere are not new [5, 10]. However, there has been a rapid discovery phase in rhizosphere microbiome research and moving now to more functionally-explicit sampling approaches will build on recent understanding by reducing experimental noise. The high functional and spatial heterogeneity within root systems, as can be found in other complex host environments (e.g., the colon) has strong implications for accurate measurement of the specificity of host-microbe interactions. While this complexity can require intricate research methods to ensure optimized measurement, there are simple ways to improve current practices while methodological research continues to advance identification of best practices (Box 1).

**Box 1:** Recommended improvements in current methodological practices and areas for methodological advancements for root sampling and rhizosphere removal methods in the rhizosphere microbiome research field

**Recommendations for root and rhizosphere sampling approaches**
1. Match sampling schemes to research questions
*Pooled sampling* net impacts on/of the rhizosphere microbiome; encompassing and coarse rhizosphere microbiome characterization
*Separated sampling* functional root-microbe relationships; targeted and variation-minimized rhizosphere microbiome characterization
2. Select functionally-explicit methods based on traits with putative functional linkage (e.g., root branching order)
3. Report how and why root sampling and rhizosphere removal methodologies were selected
4. Report in discussion how root sampling schemes may have influenced interpretation of results (e.g., false negatives)
**Research priorities for methodological advancement**
1. Simplified exudate measurement methods
2. Identification of root traits with functional links to rhizosphere microbiomes
3. Consequences of variable rhizosphere removal methods for rhizosphere microbiome characterization
4. Standardized methods and language for characterizing gradients within the rhizosphere and/or regions of the rhizosphere

## Supplementary Information

**Additional file 1**: Table of publications selected for review using “rhizosphere microbiome” search terms.

## Data Availability

All data generated and/or analyzed during this review are included in this published article and its supplementary information file.
